# TP73-AS1 as a predictor of clinicopathological parameters and prognosis in human malignancies: a meta and bioinformatics analysis

**DOI:** 10.1186/s12885-022-09658-2

**Published:** 2022-05-25

**Authors:** Caizhi Chen, Jingjing Wang, Yeqian Feng, Ye Liang, Yan Huang, Wen Zou

**Affiliations:** Department of Oncology, The Second Xiangya Hospital of Central South University, Changsha, 410000 Hunan China

**Keywords:** Lnc RNA TP73-AS1, Clinicopathological parameters, Prognosis, malignancies, Meta-analysis, Bioinformatics

## Abstract

**Background:**

Long non-coding RNA P73 antisense RNA 1 T (non-protein coding), also known as Lnc RNA TP73-AS1, is dysregulated in various tumors but the correlation between its expression and clinicopathological parameters and/or prognoses in cancer patients is inconclusive. Here, we performed a meta-analysis to evaluate the prognostic value of Lnc RNA TP73-AS1 for malignancies.

**Methods:**

We systematically searched four online databases including PubMed, the Web of Science, Embase, and the Cochrane Library for eligible articles published up to June 29/2020. Odds ratios (ORs) and Pooled hazard ratios (HRs) with 95% confidence intervals (95% CIs) were used to assess the association of TP73-AS1 expression with prognostic and clinicopathological parameters. We further validated TP73-AS1 expression in various malignancies and its potential prognostic value using the GEPIA online database. We predicted potential biological processes and relevant signal mechanisms through the public databases.

**Results:**

A total of 26 studies examining 14 cancers were analyzed to evaluate the relationship between TP73-AS1 expression, clinicopathological features and prognostic indicators. The results indicated that TP73-AS1 expression markedly correlates with TNM stage (OR = 3.27,95% CI:2.43–4.39, *P* < 0.00001), tumor size (OR = 3.00, 95%CI:2.08–4.35, *P* < 0.00001), lymph node metastasis (OR = 2.77, 95%CI:1.42–5.38,*P* < 0.00001) and distant metastasis (OR = 4.50,95%CI:2.

62–7.73,*P* < 0.00001). No correlation with age (OR = 1.12,95%CI:0.77–1.64, *P* > 0.05), gender (OR = 1.08, 95%CI:0.84–1.38, *P* > 0.05) or differentiation (OR = 1.39, 95%CI:0.71–2.70, *P* = 0.340) was observed. TP73-AS1 overexpression was a biomarker of poor Overall survival(OS)(HR = 1.85,95%CI:1.53–2.22, *P* < 0.00001) and Disease-Free-Survival (DFS) (HR = 1.57,95%CI:1.03–2.42, *P* < 0.05). Dysregulated TP73-AS1 expression and its prognostic value in various cancers was validated based on The Cancer Genome Atlas (TCGA). Further biological function predictions indicated that TP73-AS1 was involved in pro-oncogenic signaling.

**Conclusions:**

The upregulation of Lnc RNA TP73-AS1 was related to detrimental clinicopathological parameters and can be considered an indicator of poor prognosis for cancer malignancies.

## Introduction

Cancer is a global health problem with increasing morbidity and mortality with an additional economic burden to patients worldwide. According to recent cancer statistics, 1,762,450 new cancer cases and 606,880 cancer-related deaths occurred in the United States in 2019 [1]. Medical advances have allowed for the standardization of tumor treatments that can delay disease progress. However, patients with advanced cancers have a poor prognosis and quality of life. New cancer markers to enable early diagnosis and prognosis predictions are therefore urgently required.

Long non-coding RNAs (lncRNAs) were originally regarded as transcriptional noise and are defined as RNA transcripts greater than 200 nucleotides that do not encode proteins [2]. LncRNAs have been found to play crucial roles in modulating diverse aspects of tumorigenesis, including cell growth, invasion, metastasis and survival [3, 4]. Overexpression of the lncRNA TUC338 correlates with poor OS in prostate carcinoma [5]. Furthermore, the upregulation of LINC00520 is associated with a poor prognosis in patients with NPC [6]. LncRNAs therefore participate in tumorigenesis and can be used to predict the prognosis of malignancies.

TP73-AS1, located at region lp36.32, is a newly discovered lncRNA, also termed PDAM or KIAA0495 [7]. LncRNA TP73-AS1 expression is dysregulated in various human malignancies, including hepatocellular carcinoma, osteosarcoma and gastric cancer. Furthermore, previous studies indicate a correlation between abnormal TP73-AS1 expression and clinicopathological features and prognoses. However, owing to the small number of clinical patients studied, the significance of these associations is poorly characterized. Larger sample sizes are required.

In this study, we evaluated the utility of lncRNA TP73-AS1 as a prognostic cancer biomarker. A meta-analysis of all correlative studies of lncRNA TP73-AS1 and cancer was performed to assess the association between TP73-AS1 expression and clinicopathological characteristics including age, gender, TNM stage, tumor size, lymph node metastasis, distant metastasis, and differentiation. Our ultimate aim was to assess the value of lncRNA TP73-AS1 as an indicator of survival for different malignancies.

## Methods and materials

### Search strategy

We searched PubMed, Web of Science, Embase, and the Cochrane Library using the search terms “PDAM” OR “TP73-AS1” OR “KIAA0495” OR “PDAM lncRNA” OR “TP73-AS1 lncRNA” OR “KIAA0495 lncRNA” OR “lncRNA KIAA0495” OR “lncRNA PDAM” OR “lncRNA TP73-AS1” OR “long noncoding RNA TP73-AS1” and “Neoplasia” OR “Neoplasia’s” OR “Neoplasm” OR “Tumors” OR “Tumor” OR “Cancer” OR “Cancers” OR “Malignancy” OR “Malignancies” OR “Malignant Neoplasms” OR “Malignant Neoplasm” OR “Neoplasm, Malignant” OR “Neoplasms, Malignant” OR “Benign Neoplasms” OR “Neoplasms, Benign” OR “Benign Neoplasm” OR “Neoplasm, Benign” as keywords to identify all relevant publications. The search included manuscripts published up until June 29^th^ 2020. Only articles written in English were included.

### Inclusion and exclusion criteria

Inclusion criteria were as follows: (1) original primary research articles; (2) elucidated associations between TP73-AS1 expression and clinicopathological characterization or prognosis of various solid human malignancies; (3) division of patients into two groups according to TP73-AS1 expression from tissue sample analysis; (4) expression detectable by qRT-PCR; (5) reported data or Kaplan–Meier curves available for calculation of the hazard ratios (HR) (95% CI) for survival.

Exclusion criteria were as follows: (1) non-primary research articles, including case reports, reviews, conference abstracts, or letters; (2) duplicate studies; (3) studies lacking sufficient data or p-values to calculate the HR (95% CI); (4) nonhuman studies.

### Data extraction and quality assessments

Studies were selected by two reviewers based on the inclusion and exclusion criteria. Disagreements were resolved by a third reviewer. The following information was collected from each study: author last name, publication year, country, sample type and detection method, criteria for patient categorization based on TP73-AS1 expression level, the hazard ratio (HR), corresponding 95% confident interval (CI) of overall survival (OS), disease free survival (DFS) and follow-up time. The HR (95% CI) of survival was obtained directly from the study direct or calculated using Engauge Digitizer 4.1 software if Kaplan–Meier curves were available.

The Newcastle–Ottawa quality assessment scale (NOS) that considers selection, outcome, and comparability, was used to assess the quality of the studies for inclusion. Manuscripts were given NOS scores from 0 to 9, with scores ≥ 6 showing the potential to compromise the study.

### Statistical analysis

Pooled odds ratios (ORs) with 95% confidence intervals (CIs) were used to assess potential associations between of TP73-AS1 expression and clinicopathological features. Pooled HR values (95% CI) were calculated to identify TP73-AS1 as a prognostic biomarker in tumors. I^2^ statistics and chi-square Q tests were used to evaluate heterogeneity amongst eligible studies. A Chi-squared test of P < 0.10 or I^2^ > 50% indicated heterogeneity between the studies. A fixed model was used to integrate the results at an I^2^ ≤ 50%. Otherwise, the random-effects model was selected. Sensitivity analysis was performed to investigate the stability of the results. Publication bias was evaluated through a Begg’s assessments. STATA software version 12.0 was used for all statistical analysis. Significant differences were defined as *P*-values < 0.05.

### TP73-AS1 expression for prognostic predictions

We compared the expression of TP73-AS1 in diverse tumor tissues with normal tissue, and validated its prognostic role using Gene Expression Profiling Interactive Analysis (GEPIA) (http://gepia.cancer-pku.cn/), an online database from The Cancer Genome Atlas (TCGA) (https://tcga-data.nci.nih.gov). Targetscan (http://www.targetscan.org/vert_72/), mirdb (http://mirdb.org/), mirtarbase (http://mirtarbase.mbc.nctu.edu.tw/php/index.php), mircode (http://www.mircode.org/) were used to identify TP73-AS1 relevant ceRNAs. Cytoscape software was used to construct visualized lncRNA-miRNA-mRNA networks. TP73-AS1 biological functions and cancer-related pathways were predicted according to the Kyoto Encyclopedia of Genes and Genomes (KEGG) and Gene Ontology (GO) via R package clusterprofiler.

## Results

### Search results

**Fig. 1:**
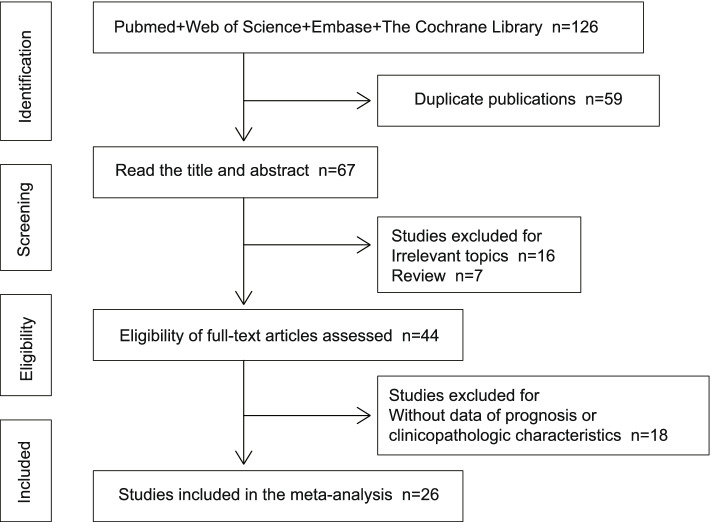
Screening process of the included studies

A total of 126 relevant publications were acquired after searching PubMed, Web of Science, Embase, and the Cochrane Library. Following the removal of duplicate publications, 67 studies were considered. Titles and abstracts were reviewed, and 16 non-relevant papers and 7 review articles were excluded. Further screening of the full texts of the 44 remaining articles led to the elimination of 18 reports due to a lack of study data regarding prognoses or clinicopathologic characteristics. Finally, 26 studies [8–33] from 2017 to 2020 met the criteria for the meta-analysis. Figure 1 describes the selection process for the included publications.

## Included studies

The characteristics of the 26 included articles are summarized in Table 1. A total of 1770 patients with sample sizes ranging from 36 to 132 were evaluated and divided into high and low TP73-AS1 expression groups according to the mean cut-off value. All studies were performed in China and published from 2017 to 2020. The studies examined a wide variety of cancers, including hepatocellular carcinoma (2 studies), osteosarcoma (2 studies), gastric cancer (5 studies), ovarian cancer (2 studies), clear cell renal cell carcinoma (1 study), bladder cancer (1 study), breast cancer (3 studies), cholangiocarcinoma (1 study), lung cancer (3 studies), brain glioma (1 study), pancreatic cancer (1 study), colorectal cancer (2 studies),cervical cancer (1 study) and retinoblastoma (1 study). The expression of TP73-AS1 in tissues was through qRT-PCR analysis. OS data were reported in 19 studies, with 3 studies reporting DFS. The NOS assessment scores of the included studies ranged from 6 to 8.

**Table 1:** Main characteristics of the studies included in the meta-analysis

**Author**	**Year**	**Country**	**Cancer**	**Sample**	**Method**	**patients number**			**OS**		**DFS**		**Follow up** **(months)**	**Data extraction method**	**NOS**	**Ref**
						**high expression**	**low expression**	**total**	**HR (95% CL)**	**P**	**HR (95% CL)**	**P**				
Li	2017	China	hepatocellular carcinoma	tissue	qRT-PCR	42	42	84	2.25(1.14–4.43)	0.019	NM	NM	25	reported	8	[8]
Chen	2018	China	osteosarcoma	tissue	qRT-PCR	66	66	132	1.90(1.15–3.13)	0.012	NM	NM	72	reported	8	[27]
Ding	2018	China	gastric cancer	tissue	qRT-PCR	38	34	72	1.19(0.47–3.03)	0.710	NM	NM	60	K-M	7	[11]
Li	2018	China	ovarian cancer	tissue	qRT-PCR	36	26	62	2.16(1.05–4.46)	0.036	NM	NM	60	K-M	7	[28]
Liu	2018	China	ccRCC	tissue	qRT-PCR	24	16	40	1.00(0.10–9.97)	0.999	1.20(0.21–6.81)	0.840	50	K-M	6	[15]
Peng	2018	China	gastric cancer	tissue	qRT-PCR	27	31	58	2.49(1.06–5.83)	0.036	NM	NM	60	K-M	7	[12]
Tuo	2018	China	bladder cancer	tissue	qRT-PCR	64	64	128	0.79(0.24–2.63)	0.706	0.83(0.37–1.84)	0.639	60	K-M	7	[16]
Wang	2018	china	ovarian cancer	tissue	qRT-PCR	30	30	60	NM	NM	NM	NM	NM	NM	6	[17]
Wang	2018	China	gastric cancer	tissue	qRT-PCR	30	34	64	1.82(1.09–3.04)	0.022	2.14(1.26–3.63)	0.005	70	K-M	7	[13]
Yang	2018	China	osteosarcoma	tissue	qRT-PCR	23	23	46	1.92(0.71–5.18)	0.199	NM	NM	50	K-M	6	[10]
Yao	2018	China	breast cancer	tissue	qRT-PCR	18	18	36	3.34(1.03–10.82)	0.045	NM	NM	50	reported	8	[18]
Yao	2018	China	cholangiocarcinoma	tissue	qRT-PCR	41	34	75	NM	NM	NM	NM	NM	NM	6	[29]
Zhang	2018	China	NSCLC	tissue	qRT-PCR	22	23	45	1.76(0.65–4.74)	0.267	NM	NM	60	K-M	6	[21]
Zhang	2018	China	brain glioma	tissue	qRT-PCR	24	23	47	2.46(1.13–5.35)	0.023	NM	NM	40	reported	8	[24]
Zhang	2018	China	gastric cancer	tissue	qRT-PCR	41	35	76	1.42(0.72–2.81)	0.310	NM	NM	60	K-M	6	[14]
Zou	2018	China	breast cancer	tissue	qRT-PCR	43	43	86	NM	NM	NM	NM	NM	NM	6	[19]
Cui	2019	China	pancreatic cancer	tissue	qRT-PCR	45	32	77	2.14(1.18–3.87)	0.012	NM	NM	50	K-M	7	[25]
Jia	2019	China	colorectal cancer	tissue	qRT-PCR	30	31	61	NM	NM	NM	NM	NM	NM	8	[26]
Liu	2019	China	lung adenocarcinoma	tissue	qRT-PCR	37	43	80	1.60(0.45–5.71)	0.467	NM	NM	60	K-M	6	[23]
Ma	2019	China	hepatocellular carcinoma	tissue	qRT-PCR	30	30	60	NM	NM	NM	NM	NM	NM	6	[9]
Zhang	2019	China	cervical cancer	tissue	qRT-PCR	NM	NM	56	2.37(0.75–7.50)	0.142	NM	NM	60	K-M	7	[20]
Zhu	2019	China	NSCLC	tissue	qRT-PCR	33	39	72	0.88(0.28–2.76)	0.825	NM	NM	60	K-M	8	[22]
Li	2019	China	colorectal cancer	tissue	qRT-PCR	33	37	70	1.31(0.59–2.92)	0.510	NM	NM	60	K-M	7	[32]
Liu	2020	China	gastric cancer	tissue	qRT-PCR	34	34	68	NM	NM	NM	NM	NM	NM	6	[30]
Liu	2020	China	breast cancer	tissue	qRT-PCR	25	20	45	NM	NM	NM	NM	NM	NM	6	[31]
Wang	2020	China	retinoblastoma	tissue	qRT-PCR	37	33	70	2.72(0.85–8.68)	0.090	NM	NM	60	K-M	7	[33]

Abbreviations: *OS* overall survival; *DFS* disease-free survival; *HR* hazard ratio; *CI* confidence interval; *qRT-PCR* quantitative reverse transcription polymerase chain reaction; *NM* not mentioned; *K-M* Kaplan–Meier plot; *ccRCC* Clear Cell Renal Cell Carcinoma; *NSCLC* non-small cell lung cancer; *NOS* Newcastle–Ottawa Scale, *Ref* reference

## Correlation between TP73-AS1 and clinicopathologic parameters

### Relationship between TP73-AS1 expression and age

**Fig. 2:**
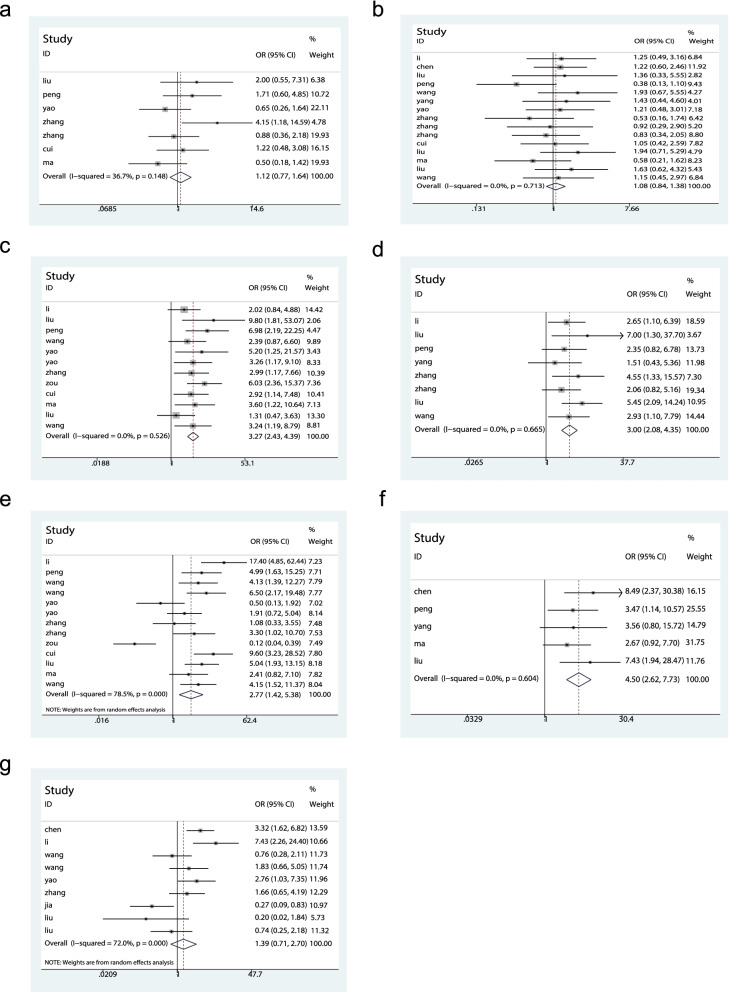
Forest plots assessing the correlation between TP73-AS1 expression and clinicopathological parameters. **a** Age. **b** gender. **c** TNM stage. **d** tumor size. **e** lymph node metastasis. **f** distant metastasis and **g** differentiation

As shown in Fig. 2a, seven studies explored the correlation between TP73-AS1 expression and age. No noticeable heterogeneity was observed amongst the studies (I^2^ = 36.7%, P = 0.148) so a fixed model was employed for analysis. No significant correlation was identified between TP73-AS1 overexpression and patient age (OR = 1.12, 95% CI 0.77–1.64, *P* > 0.05).

### Relationship of TP73-AS1 expression with gender

Thirteen studies investigated potential associations between TP73-AS1 expression and gender. Upon assessment with the fixed model, no heterogeneity was observed amongst the studies (I^2^ = 0%, *P* = 0.713). As shown in Fig. 2b, expression of TP73-AS1 did not correlate with patient gender (OR = 1.08, 95% CI 0.84–1.38, *P* > 0.05).

### Relationship of TP73-AS1 with TNM stage

TNM stage and TP73-AS1 expression were reported for 794 patients across twelve studies. The pooled results showed an OR = 3.27 (95% CI: 2.43–4.39, *P* < 0.00001) with no notable heterogeneity (I^2^ = 0%, *P* = 0.526). The data were therefore analyzed using a fixed model (Fig. 2c). The upregulation of TP73-AS1 was significantly associated with advanced TNM stage.

### Relationship of TP73-AS1 expression with tumor size

The relationship between tumor size and TP73-AS1 expression was evaluated for 8 studies of 501 patients. Forest plots indicated no evident of heterogeneity amongst the studies (I2 = 0%, *P* = 0.665). Subsequent analysis indicated that the overexpression of TP73-AS1 correlated with a tumor size ≧ 5 cm (OR = 3.00, 95% CI: 2.08–4.35, *P* < 0.00001) (Fig. 2(d)).

### Relationship between TP73-AS1 expression and lymph node metastasis

Data for total OR and 95% CI of LNM were collected from 13 studies. Data analysis yielded a pooled OR of 2.77 (95% CI 1.42–5.38, *P* < 0.00001) using a random model, owing to significant heterogeneity (I^2^ = 78.5%, P ≤ 0.001) (Fig. 2e). We concluded that TP73-AS1 overexpression was associated with lymph node metastasis of cancer.

### Relationship between TP73-AS1 expression and distant metastasis

Five studies involving 341 patients were analyzed to evaluate the correlation between TP73-AS1 expression and distant metastasis. No significant heterogeneity was detected amongst the studies(I^2^ = 0%, *P* = 0.604, fixed-model). Model results indicated that TP73-AS1 upregulation was related to distant metastasis (OR = 4.50, 95% CI: 2.62–7.73, *P* < 0.00001), (Fig. 2f).

### Relationship between TP73-AS1 expression and differentiation

As shown in Fig. 2g, eight studies were used to evaluate the correlation between TP73-AS1 expression and histological tumor differentiation. The random model was performed owing to heterogeneity amongst the studies (I^2^ = 72.0%, *P* ≤ 0.001) with a pooled OR = 1.39 (95% CI: 0.71–2.70, *P* = 0.340). No marked differences were detected in differentiation status between the two groups.

## Association between TP73-AS1 and prognostic indicators

### Relationship of TP73-AS1 expression with OS

**Fig. 3:**
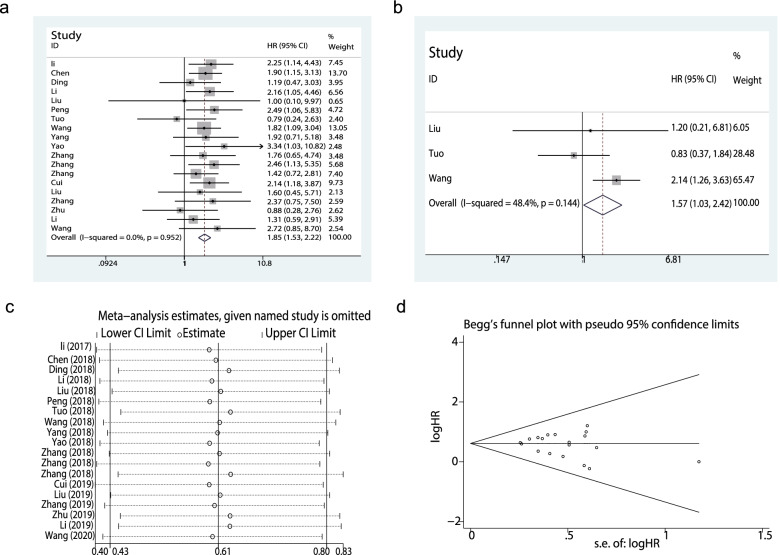
Forest plots assessing. **a** the correlation between TP73-AS1 expression and overall survival (OS). **b** TP73-AS1 expression and disease-free survival (DFS). **c** sensitivity analysis for OS; and. **d** Begg’s assessments of OS

A total of 19 studies with data from 1315 patients were used to determine the utility of TP73-AS1 as a prognostic biomarker of cancer based on OS data. Pooled HR = 1.85 (95% CI: 1.53–2.22, *P* < 0.00001) (Fig. 3a). A fixed-effects model was used to estimate the HR of the studies, that showed no apparent heterogeneity (I^2^ = 0%, *P* = 0.952). These data indicated that the upregulation of TP73-AS1 was associated with a poorer OS amongst multiple types of malignancies.

### Relationship of TP73-AS1 expression with DFS

Only three studies reported DFS data that could be used to assess the prognostic effects of TP73-AS1. Our analysis suggested that TP73-AS1 overexpression was associated with DFS (pooled HR: 1.57, 95% CI: 1.03–2.42, *P* < 0.05), (Fig. 3b). No obvious heterogeneity was observed amongst the studies (I^2^ = 48.4%, *P* = 0.144).

## Sensitivity analysis and publication bias

A sensitivity analysis was used to evaluate the robustness of the pooled results. The data were deemed reliable without the removal of any studies (Fig. 3c). No publication bias was observed through Begg’s tests (Fig. 3d, *P* = 0.368).

## Subgroup analysis of OS

**Fig. 4:**
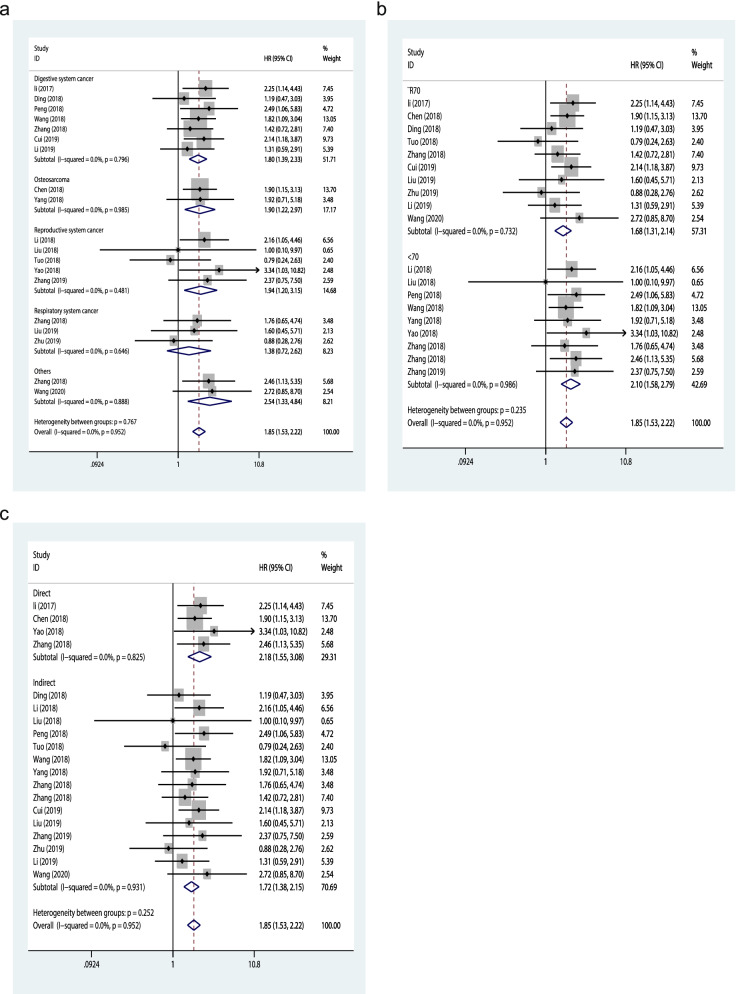
Forest plots for the subgroup analysis of OS. **a** cancer type; **b **sample size; **c** extracted method

Subgroup analysis was performed in terms of cancer types, sample sizes and data extraction methods to further analyze the predictive value of TP73-AS1 (Fig. 4a–c, Table 2). As for different type of cancers, promoted TP73-AS1 expression level was closely associated with worse OS of the patients with digestive system cancers (HR = 1.80, 95%CI:1.39–2.33, *P* = 0.000), osteosarcomas (HR = 1.90, 95% CI:1.22–2.97, *P* = 0.005), respiratory system cancers (HR = 1.94, 95% CI:1.20–3.15, *P* = 0.007) and other cancers (HR = 2.54, 95% CI:1.33–4.84, P = 0.005), except for reproductive system cancers (HR = 1.38, 95% CI:0.72–2.62, *P* = 0.334). In the stratified analysis by sample sizes, we found that TP73-AS1 upregulation significantly related to unfavorable OS in the studies with sample size ≧70 (HR = 1.68, 95% CI:1.31–2.14, *P* = 0.000), as well as those with sample size < 70 (HR = 2.10, 95% CI:1.58–2.79, *P* = 0.000). When the studies were categorized according to different data extraction methods, the subgroup analysis demonstrated that the prognostic value of TP73-AS1 on the OS was not influenced by data extraction methods, that is, the HR provided in the research (HR = 2.18, 95% CI:1.55–3.08, *P* = 0.000) or extracted from the K-M curves (HR = 1.72, 95% CI:1.38–2.15, *P* = 0.000). No evident heterogeneity was found within the subgroups.

**Table 2:** Subgroup meta-analysis of the association between TP73-AS1 expression and OS

Subgroup	Studies	HR	95%CI	*P* value	Model	Heterogeneity	
						**I2**	***P* Value**
Cancer type Digestive system cancer	7	1.80	1.39–2.33	0.000	Fixed	0%	0.796
Osteosarcoma	2	1.90	1.22–2.97	0.005	Fixed	0%	0.985
Respiratory system cancer	3	1.94	1.20–3.15	0.007	Fixed	0%	0.481
Reproductive system cancer	5	1.38	0.72–2.62	0.334	Fixed	0%	0.646
Others	2	2.54	1.33–4.84	0.005	Fixed	0%	0.888
Sample size ≧70	10	1.68	1.31–2.14	0.000	Fixed	0%	0.732
< 70	9	2.10	1.58–2.79	0.000	Fixed	0%	0.986
Extracted method							
Direct	4	2.18	1.55–3.08	0.000	Fixed	0%	0.825
Indirect	15	1.72	1.38–2.15	0.000	Fixed	0%	0.931

*OS* overall survival; *HR* hazard ratio; *95% CI* 95% confidence interval

## Verification of TP73-AS1 expression and its prognostic value based on the TCGA

**Fig. 5:**
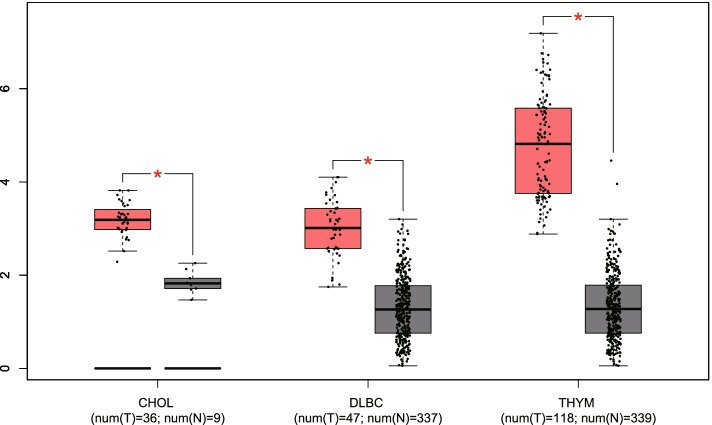
TP73-AS1 expression in three types of cancer *vs.* normal tissue. “*”丨Log_2_FC丨 > 1 and *P* < 0.01. Abbreviations: CHOL: Cholangiocarcinoma; DLBC: Diffuse Large B-cell Lymphoma; THYM: Thymoma

**Fig. 6:**
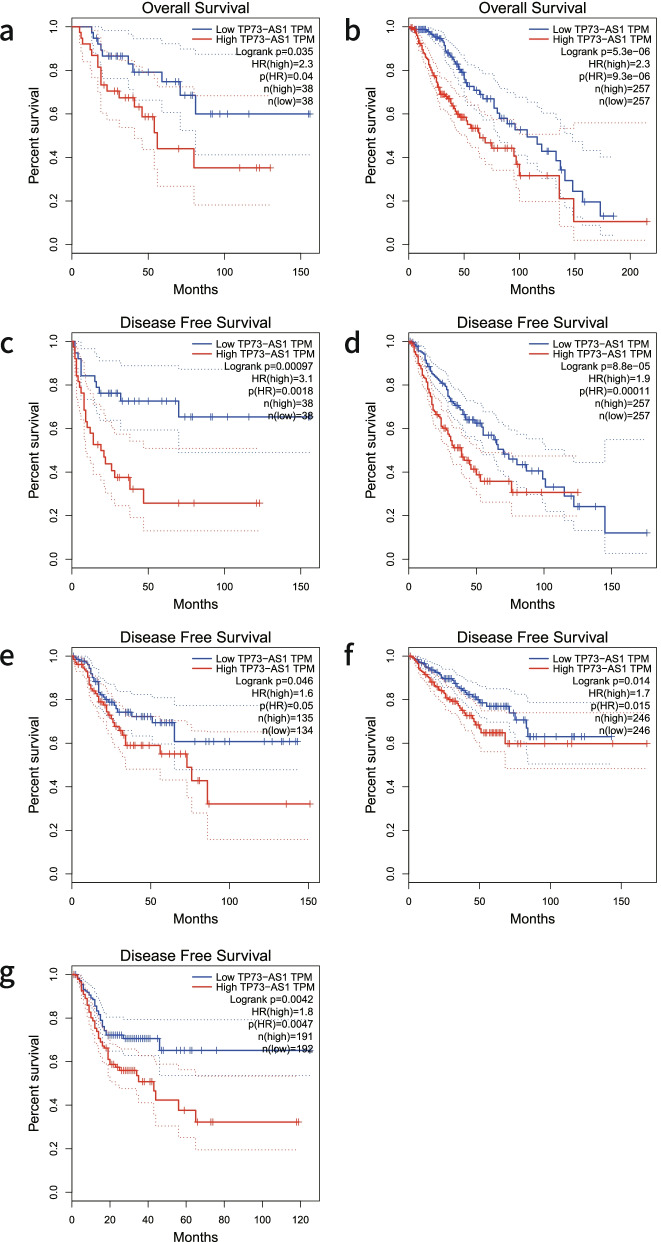
Verification of the prognostic value of TP73-AS1 in the TCGA database. **a** OS plots of TP73-AS1 in ACC. **b** OS plots of TP73-AS1 in LGG. **c** DFS plots of TP73-AS1 in ACC. **d** DFS plots of TP73-AS1 in LGG. **e** DFS plots of TP73-AS1 in COAD. **f** DFS plots of TP73-AS1 in PRAD. **g** DFS plots of TP73-AS1 in STAD. Abbreviations: TCGA: The Cancer Genome Atlas; ACC: Adenocarcinoma; Carcinoma; LGG: Brain Lower Grade Glioma; COAD: Colon Adenocarcinoma; PRAD: Prostate Adenocarcinoma; STAD: Stomach Adenocarcinoma

To validate the expression of TP73-AS1 in diverse cancers, we used the GEPIA online tool for gene analysis. As shown in Fig. 5, the expression of TP73-AS1 was dramatically upregulated in three malignancies including cholangiocarcinoma, lymphoid neoplasm diffuse large B-cell lymphoma and thymoma (|Log2fold change (FC)| cutoff > 1 and *P* < 0.01). In addition, log-rank analysis and Kaplan–Meier curves were used to verify the association between TP73-AS1 expression and the prognostic index of patients with different malignancies. Similar to our meta-analysis, the overexpression of TP73-AS1 correlated to a poorer OS in adrenocortical carcinoma (ACC) and low grade glioma (LGG) (log-rank *P* < 0.05) (Fig. 6a–b). Moreover, the upregulation of TP73-AS1 was associated with a poorer DFS in adrenocortical carcinoma (ACC), low grade glioma (LGG), colon adenocarcinoma (COAD), prostate adenocarcinoma (PRAD) and stomach adenocarcinoma (STAD) (log-rank *P* < 0.05) (Fig. 6c-g). These results indicated that TP73-AS1 serves as a novel prognostic biomarker for cancer malignancies.

## Prediction of TP73-AS1 function

**Fig.7:**
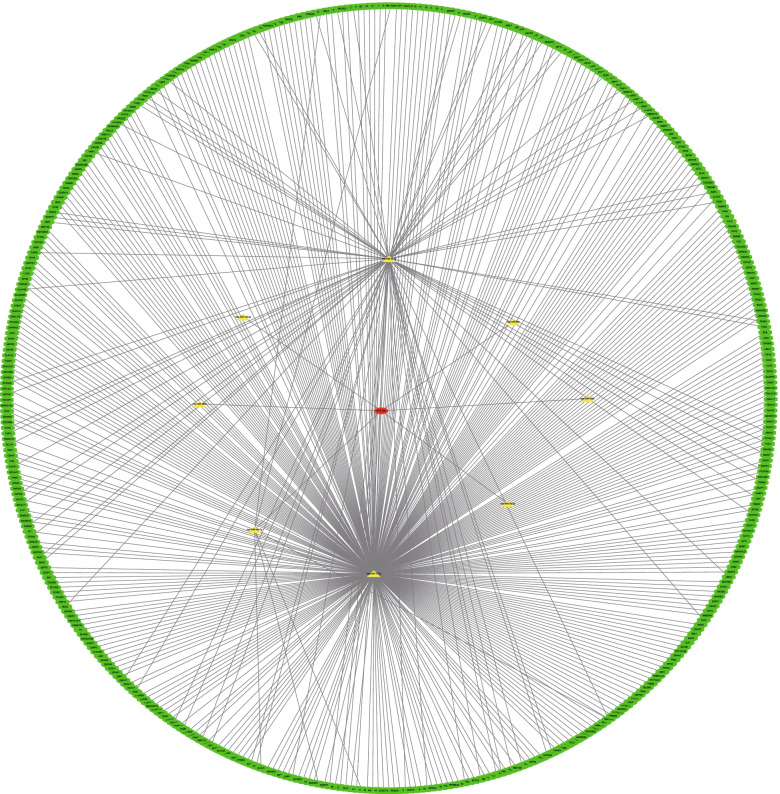
Establishment of TP73-AS1-mediated ceRNA net. TP73-AS1-mediated ceRNA networks including 8 miRNAs and 448 mRNAs. Green octagons represent mRNAs; yellow triangles represent miRNAs; red ovals represent TP73-AS1

**Fig. 8:**
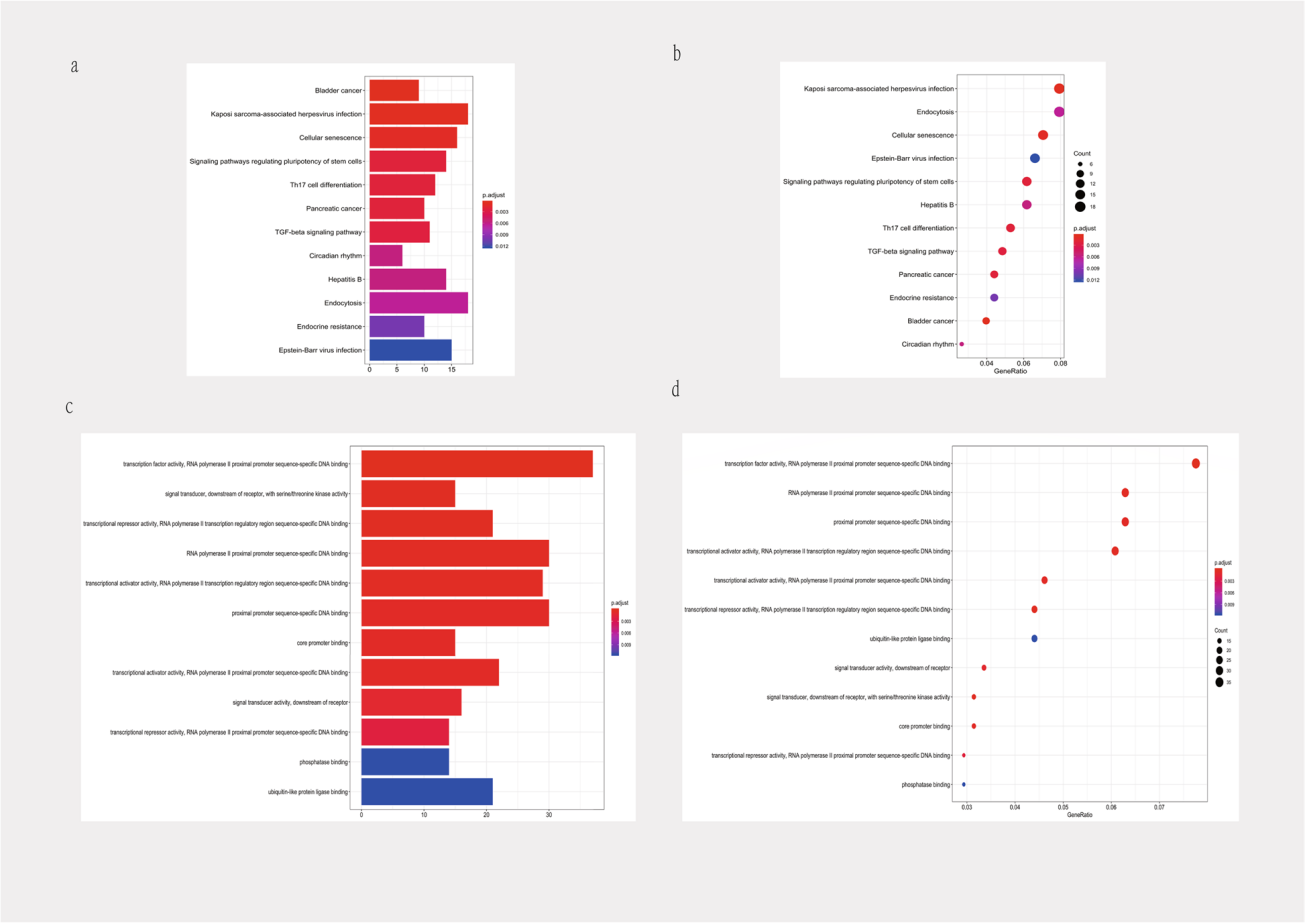
KEGG and GO term enrichment for TP73-AS1. **a** Barplots of KEGG molecular mechanisms. **b** dotplots of KEGG molecular mechanisms. **c** barplots of GO enrichment. **d** dotplots of GO enrichment

We predicted potential biological functions and the molecular mechanisms of TP73-AS1 in cells using public databases. We first explored ceRNA modulations for TP73-AS1 using targetscan, mirdb, mirtarbase, and mircode databases. TP73-AS1-miRNA-mRNA networks containing 8 miRNAs and 448 mRNA were constructed using cytoscape (Fig. 7). Figure 8 shows the top 12 KEGG and Go pathways. TP73-AS1 was predicted to participate in tumor signaling, including Kaposi sarcoma-associated herpesvirus infection, signaling pathways regulating the pluripotency of stem cells and TGF-beta signaling (Fig. 8a-b). GO functional enrichment analysis indicated that the molecular functions of TP73-AS1 included RNA polymerase II proximal promoter sequence-specific DNA binding, proximal promoter sequence-specific DNA binding, and core promoter binding (Fig. 8c-d).

## A flow diagram of the meta-analysis and bioinformatics analysis

**Fig. 9:**
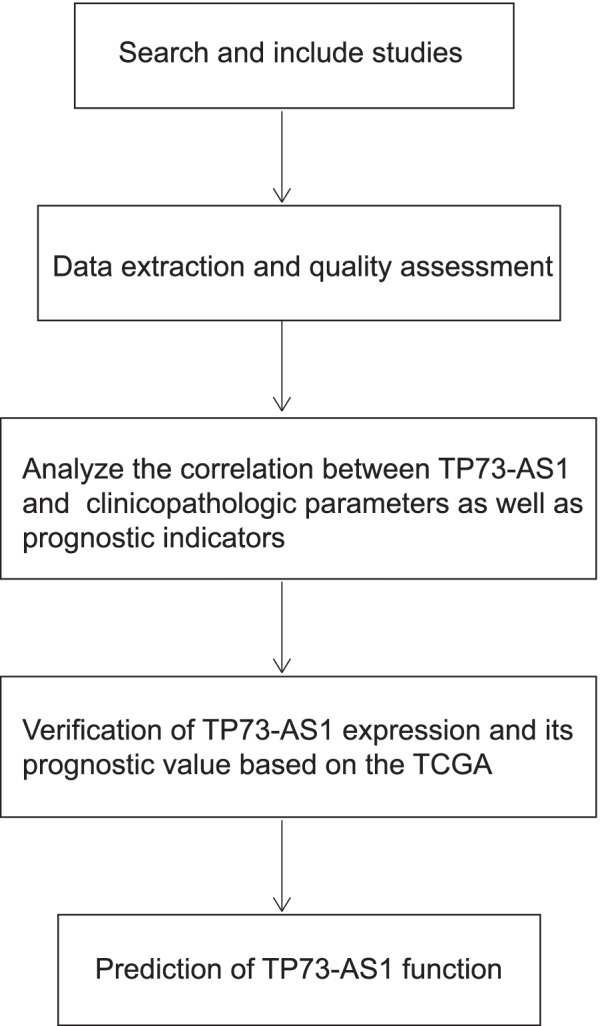
A flow diagram of the meta-analysis and bioinformatics analysis

In our paper, we first searched and selected studies met inclusion criteria and then extracted data from the papers.Thirdly, we analyzed the correlation between TP73-AS1 and clinicopathology of patients as well as prognostic indicators.Finally, we verificated TP73-AS1 expression and its prognostic value based on the TCGA and predicted its functions.The whole flow diagram was showed in Fig. 9.

## Discussion

LncRNA TP73-AS1 is dysregulated in many tumor types. A meta-analysis was performed to examine the association between TP73-AS1 expression, clinicopathological features and prognostic values in patients. A total of twenty six studies examining 1770 patients and 14 cancers were included. The results suggested that the upregulation of TP73-AS1 significantly correlates with TNM stage, tumor size, lymph node metastasis and distant metastasis but not with the age, gender or tumor differentiation (Table 3). Furthermore, we found that TP73-AS1 overexpression is associated with poor OS and DFS, indicating that TP73-AS1 is an effective biomarker for the diagnoses and prognoses of malignancies. To further strengthen our conclusions, all finding were validated using the GEPIA database. TP73-AS1 was found to be overexpressed in CHOL, DLBC and THYM. Moreover, the OS of patients was lower in patients with high TP73-AS1 expression in ACC and LGG. Similar results were observed for the DFS of patients with ACC, LGG, COAD, PRAD and STAD.

**Table 3:** Results of the association between TP73-AS1 and clinicopathological parameters

Outcome	Studies	OR	95%CI	*P* value	Model	Heterogeneity	
						**I2**	***P* Value**
Age (< 60 vs ≥ 60)	7	1.12	0.77–1.64	0.545	Fixed	36.7%	0.148
Gender (male vs female)	15	1.08	0.84–1.38	0.560	Fixed	0%	0.713
TNM stage (III-IV vs I-II)	12	3.27	2.43–4.39	< 0.00001	Fixed	0%	0.526
tumor size (≥ 5 cm vs < 5 cm)	8	3.00	2.08–4.35	< 0.00001	Fixed	0%	0.665
Lymph node metastasis (positive vs negative)	13	2.77	1.42–5.38	0.003	Random	78.5%	≤ 0.001
distant metastasis (yes vs no)	5	4.50	2.62–7.73	< 0.00001	Fixed	0%	0.604
Differentiation (poor vs well)	9	1.39	0.71–2.70	0.340	Random	72.0%	≤ 0.001

Previous studies investigated the molecular mechanisms of TP73-AS1 in diverse tumors, regarding cell proliferation, invasion, metastasis, and apoptosis. In hepatocellular carcinoma, TP73-AS1 promotes cell growth via a TP73-AS1/miR-200a/HMGB1/RAGE signaling axis and accelerates malignant progression through regulating microRNA-103 [8, 9]. In osteosarcoma, Yang et al. [10] found that TP73-AS1 silencing suppressed proliferation and invasion which when combined with miR-142 could avoid Rac1 binding. In gastric cancer, the downregulation of TP73-AS1 suppressed cell proliferation, invasion and migration via miR-194-5p/SDAD1, HMGB1/RAGE, WNT/β catenin or EMT/Bcl-2/caspase-3 signaling pathways but Liu et al. found that miR-223-5p may target TP73-AS1 to promote the invasion and migration of gastric cancer patients [11–14, 30]. In clear cell renal cell carcinoma, TP73-AS1 silencing suppressed cell proliferation and promoted apoptosis in vitro through the upregulation of KISS1 expression and the activation of PI3K/Akt/mTOR signaling [15]. In bladder cancer, Tuo et al. [16] showed that TP73-AS1 suppressed EMT. In the female reproductive system, Wang et al. [17] demonstrated that TP73‐AS1 facilitated cell proliferation and the metastasis of ovarian cancer through its regulation of MMP2 and MMP9 expression. In addition, TP73-AS1 boosted cell proliferation, invasion, and migration, and functioned as a ceRNA with miR-200a to prevent binding of TFAM, dampening the TP73-AS1/miR-200a/ZEB1 and TP73-AS1/miRNA-125a-3p/ metadherin axis in breast cancer [18, 19, 31]. Zhang et al. [20] also reported that the upregulation of TP73-AS1 promotes the tumorigenesis of cervical cancer by promoting CCND2 through the suppression of miR-607 expression. In the respiratory system, TP73-AS1 promotes tumor growth and cell cycle progression of NSCLC via a pathway involving TP73-AS1/miR-449a/EZH2 or through the regulation of miR-21 which controls the progression of lung adenocarcinoma through the PI3K/AKT axis [21–23]. We further explored the mechanisms of TP73-AS1 expression in the glioma, pancreatic cancer, colorectal cancer and retinoblastoma, and similar results were observed [24–26, 32, 33]. These studies demonstrated that TP73-AS1 can play an oncogenic role in addition to its role as a tumor suppressor, dependent on the cancer type. The molecular mechanisms governing the effects of TP73-AS1 in various malignancies are summarized in Table 4.

**Table 4:** Summary of TP73-AS1 with their related signaling pathways

Study	Cancer	Aberrant expression	Biological functions	Related signaling pathways
Li 2017 [8]	hepatocellular carcinoma	upregulation	promote cell proliferation	miR-200a/HMGB1/RAGE
Chen 2018 [27]	osteosarcoma	upregulation	promote cell proliferation, migration and invasion	
Ding 2018 [11]	gastric cancer	upregulation	promote cell growth and metastasis	miR-194-5p/SDAD1
Li 2018 [28]	ovarian cancer	upregulation	promote cell proliferation	
Liu 2018 [15]	clear cell renal cell carcinoma	upregulation	promote cell proliferation, inhibit cell apoptosis	KISS/EZH2, PI3K/Akt/mTOR
Peng 2018 [12]	gastric cancer	upregulation	promote cell proliferation	HMGB1/RAGE
Tuo 2018 [16]	bladder cancer	downregulation	inhibit cell growth, cell cycle, migration and invasion, induce cell apoptosis	EMT
Wang 2018 [17]	ovarian cancer	upregulation	promoted cell proliferation, invasion, and migration	MMP2, MMP9
Wang 2018	gastric cancer	upregulation	promote cell proliferation, invasion	WNT/β-catenin
Yang 2018 [10]	osteosarcoma	upregulation	promote cell proliferation, invasion	miR-142/Rac1
Yao 2018 [18]	breast cancer	upregulation	promote cell proliferation	miR-200a/TFAM
Yao 2018 [6]	cholangiocarcinoma	upregulation	promote cell proliferation, migration, invasion, inhibit cell apoptosis	
Zhang 2018 [21]	non-small cell lung cancer	upregulation	promote cell proliferation, tumor growth and cycle progression	miR-449a/EZH2
Zhang 2018 [24]	brain glioma	upregulation	promote cell proliferation and invasion	miR-142/HMGB1/RAGE
Zhang 2018 [14]	gastric cancer	upregulation	promote cell migration and invasion	EMT/Bcl-2/caspase-3
Zou 2018 [19]	breast cancer	upregulation	promote cell invasion and migration	miR-200a/ZEB1
Cui 2019 [25]	pancreatic cancer	upregulation	promote migration and invasion	miR-141-3p/BDH2
Jia 2019 [26]	colorectal cancer	downregulation	inhibite cell growth, promote apoptosis	miR-103/ PTEN
Liu 2019 [23]	Lung adenocarcinoma	upregulation	promote cell proliferation, migration, invasion, inhibit apoptosis	PI3K/AKT
Ma 2019 [9]	hepatocellular carcinoma	upregulation	promote cell proliferation, inhibit apoptosis	miR-103
Zhang 2019 [20]	cervical cancer	upregulation	promote cell proliferation, migration and invasion	miR-607/cyclin D2
Zhu 2019 [22]	non-small cell lung cancer	upregulation	promote cell proliferation. migration and invasion	miR-21
Li 2019 [32]	colorectal cancer	upregulation	promote cell migration and invasion	TGF-β1
Liu 2020 [30]	gastric cancer	downregulation	inhibit cell invasion and migration	miR-223-5p
Liu 2020 [31]	breast cancer	upregulation	promote cell proliferation, migration and invasion, inhibite apoptosis	miRNA-125a-3p/metadherin
Wang 2020 [33]	retinoblastoma	upregulation	promote cell proliferation, metastasis and invasion	miRNA-874-3p / TFAP2B

LncRNAs indirectly modulate the function and expression of downstream genes through ceRNAs. We therefore constructed a TP73-AS1-mediated competing endogenous RNA network to evaluate possible functional and signaling pathways of TP73-AS1 in tumors. As shown in the network, TP73-AS1 could bind to eight miRNAs targeting more than 400 mRNAs. Furthermore, the predicted KEGG pathways indicated that TP73-AS1 regulates the cell proliferation, migration, invasion and apoptosis of malignancies via Kaposi sarcoma-associated herpesvirus infection, signaling pathways regulating the pluripotency of stem cells and TGF-beta signaling.

Some limitations of the study should be noted. First, all included studies were from China, so the results may only be applicable to Chinese or Asian populations. Supplementary analysis using the GEPIA database was used to compensate for this racial limitation. Secondly, a larger sample size and different tumor types are required to confirm our overall conclusions due to the limited sample size of limited number of carcinomas assessed. Thirdly, HR and 95% CI values of 15 studies were calculated from Kaplan–Meier curves. These values were likely to be less accurate than those obtained from direct measurements. Finally, we only predicted the biological functions and molecular mechanisms of TP73-AS1 ceRNA networks and utilized KEGG and GO enrichment analysis. Other broader regulatory mechanisms should now be investigated. Taken together, further studies should focus on other types of cancer, including hematological tumors with larger sample sizes to explore other potential functions of TP73-AS1 and its role during prooncogenic signaling. This information may provide novel therapeutic strategies for cancer patients.

Despite these limitations, we demonstrate that TP73-AS1 overexpression correlates with advanced TNM stage, larger tumor sizes, lymph node metastasis and distant metastasis. In addition, TP73-AS1 overexpression is related to a poor prognosis, indicating its utility as a diagnostic and prognostic marker of diverse malignancies. Further studies in larger patient cohorts and an array of cancer types are now required to validate these findings.

## Conclusions

The upregulation of LncRNA TP73-AS1 correlates with detrimental clinicopathological features and can be considered an indicator of poor prognosis for malignancies in a clinical setting.

## Data Availability

All data are included in our paper.
